# Multidisciplinary Prehabilitation Reduces Hospitalization Time and Suggests Improved Survival in Patients with Radiologically Diagnosed Lung Cancer

**DOI:** 10.3390/cancers17203329

**Published:** 2025-10-15

**Authors:** Iain Phillips, Caleb McDougall, Abi Walton, Mark Stares, Peter Hall, Robert Grecian, Fiona O’Brien, Julie Mencnarowski, Fiona MacCormick, Gavin McLean, Steven Higgins, Debbie McMillan, Colette Reid, Lindsey Allan, Benjamin Jia Liang Lim, Colin Barrie

**Affiliations:** 1 Edinburgh Cancer Centre, Western General Hospital, Edinburgh EH4 2XU, UK; caleb.mcdougall@nhs.scot (C.M.); abigail.walton@nhs.scot (A.W.); mark.stares@ed.ac.uk (M.S.); p.s.hall@ed.ac.uk (P.H.); julie.mencnarowski@nhs.scot (J.M.); colette.reid@nhs.scot (C.R.); colin.barrie@nhs.scot (C.B.); 2 St John’s Hospital, Livingston EH54 6PP, UKfiona.obrien@nhs.scot (F.O.); fiona.maccormick@nhs.scot (F.M.); gavin.mclean@nhs.scot (G.M.); steven.higgins@nhs.scot (S.H.); debbie.mcmillan@nhs.scot (D.M.); 3 Department of Nutrition and Dietetics, Royal Surrey NHS Foundation Trust, Guildford GU2 7XX, UK; lindseyallan@nhs.net; 4 Edinburgh Medical School, University of Edinburgh Medical School, Edinburgh EH16 4UU, UK; b.j.l.lim@sms.ed.ac.uk

**Keywords:** prehabilitation, lung cancer, hospital admissions, supportive care, palliative care, cachexia

## Abstract

Background/Objectives: Lung cancer is the commonest cause of cancer death worldwide. Treatment efficacy is improving, but treatment rates remain low. We aimed to assess the impact of tri-modality early prehabilitation (single in-person interventions by a senior doctor in palliative/supportive care, physiotherapist and dietitian) on patients with a radiological diagnosis of lung cancer. Methods: Patients were approached as soon as they were told they had a radiological diagnosis of lung cancer. Prehabilitation took place in parallel with a patient’s investigative pathway for lung cancer. Routine data for admissions and survival was assessed retrospectively for prehabilitation patients and compared to an appropriate group of historical controls. Results: A total of 97 patients with stage 3 or 4 lung cancer underwent prehabilitation and were compared to 199 historical controls. There were no obvious statistical differences in gender or age between the cohorts. There was a trend towards reduced mean length of stay (LOS) from 7.6 days to 2.6 days in the patients who underwent all three interventions and from 7.6 days to 3 days in all comers (*p* = 0.053), with a significantly shorter admission in those who were admitted (*p* = 0.0014). There was a reduction in time spent in hospital from 486 days to 172 days per 100 patients receiving prehabilitation (a saving of 3.1 days per patient participating). Survival appeared to improve 6 months after diagnosis, at 61% in the prehabilitation cohort (95% CI: 51.8–72.0%, *p* = 0.029) vs. 47.7% in the historical controls (95% CI: 41.3–55.2%, *p* = 0.029). Life-prolonging treatment rates in stage 4 disease increased from 26% to 42% and all-comer best supportive care rates reduced from 45% to 28%. Conclusions: Early prehabilitation appears to reduce time spent in hospital. It may also improve treatment rates and short-term survival in patients being investigated for lung cancer.

## Simple Summary

Lung cancer is very common. In approximately half of patients, the cancer has spread outside the lung when patients are diagnosed. This means the cancer is incurable for many patients. Despite improvements in drug treatments, many patients are not fit enough for treatment. This paper describes a project that attempts to improve lung cancer outcomes. Patients saw a symptom control doctor, dietitian and physiotherapist for a single appointment whilst being investigated for lung cancer. This had a positive effect on the patients. It reduced the amount of time the patients spent in hospital in the first 6 weeks from the date of lung cancer first being suspected. It may have also increased the length the patients lived after being diagnosed and increased their chance of getting treatment.

## 1. Introduction

Lung cancer is the leading cause of cancer death worldwide. Patients with lung cancer often lose weight, have high nutritional needs, a high symptom burden, have multiple co-morbidities and are older in age [1,2,3,4,5].

Prehabilitation aims to strengthen patient fitness for cancer treatment. Lung cancer treatments are improving, with long-term survival possible across all stages of disease with targeted therapies [6,7,8,9,10,11]. However, treatment rates are still low. English data suggests that only 60% of fit patients (Eastern Co-operative Oncology Group Performance Status 0–2) with stage IIIB-IV receive SACT as part of their anti-cancer treatment [12].

Prehabilitation has the potential to act as a bridge to treatment and increase both treatment rates and survival. Macmillan, a UK cancer charity, defined prehabilitation as having three domains: nutrition, physical activity and psychological support [13]. Local Scottish guidelines add symptom control as a fourth domain [14]. Prehabilitation is now increasingly recognised as a central tenet of cancer care in Scotland [15,16]. Currently, there is no defined prehabilitation pathway for those living with less survivable cancers, such as lung cancer. Palliative care, physiotherapy and dietetic interventions were chosen because patients with lung cancer have a high symptom burden, as well as a need for exercise and nutritional input. Our aim was to optimise symptoms such as pain, nausea and breathlessness through expert advice as quickly as possible, in order to facilitate more effective physical activity and nutritional interventions.

The existing prehabilitation pathways were developed using a surgical model, based on a previous Enhanced Recovery After Surgery pathway (ERAS) [17]. ERAS aims to educate patients about expectations of treatment. A more formalised pathway allows optimisation of a patient’s pre-operative physical condition. Extra steps are put in place to mobilise patients more quickly, minimise pain and support their nutrition post-operatively [18]. Existing data suggests tri-modality prehabilitation before elective surgery appears to be beneficial. Combining exercise, nutrition and psychological support appears to have a moderate certainty of benefit when compared to single or dual interventions [19].

We present the EPIC project (Early Prehabilitation In lung Cancer), which aimed to integrate tri-modality prehabilitation into the lung cancer diagnostic pathway. Patients underwent early palliative/supportive care, dietetic support and physical rehabilitation from a physiotherapist, as soon as possible after a radiological diagnosis of lung cancer was made. Our aim was to reduce the time spent in hospital around the time of diagnosis, increase treatment rates and increase survival.

We previously published the first phase of the pilot project outcomes, which showed that the time spent in hospital was reduced by 76% and the average length of stay was reduced from 8 days to 2.5 days [20]. However, the early part of the project was completed during the recovery from the COVID-19 pandemic. This paper presents a more thorough analysis of a larger cohort, assessing the impact of early prehabilitation in patients with suspected lung cancer.

## 2. Materials and Methods

In the UK, patients with suspected lung cancer are initially seen and appropriately investigated by a respiratory physician. We aimed to refer patients for tri-modality prehabilitation at their new patient respiratory appointment, typically within a week of a CT scan showing radiological suspicion of lung cancer. All patients with suspected stage 3 or 4 lung cancer who were fit for further investigation were eligible. Patients who were deemed for best supportive care or were admitted to hospital from their new respiratory appointment were ineligible.

**Figure 1:**
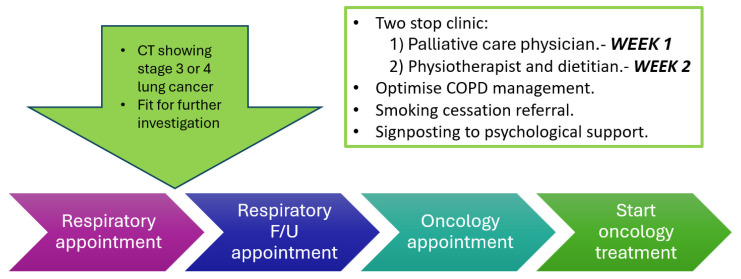
Project scheme for early intervention in patients with suspected lung cancer.

Patients saw a palliative physician in week 1 and then a physiotherapist and a dietitian in week 2 (Figure 1). This was a single in-person intervention, with the majority of patients then receiving a single telephone follow-up appointment. Patients needing more formal psychological support were referred to the local cancer support charity.

The prehabilitation project ran during 2 periods of funding. July 2021–June 2023 was funded as a joint working project between NHS Lothian and Merck, Sharpe and Dohme. Further funding from Macmillan and the Scottish Government was obtained to cover prehabilitation from March 2024 to February 2025.

A cohort of historical controls was identified for comparison. Patients diagnosed with stage 3 and 4 lung cancer from January 2019 to June 2021 and July 2023 to February 2024 were identified as potential controls. Their case files were reviewed individually for the following criteria: patients were seen in an outpatient respiratory clinic as a new patient, had a radiological diagnosis of stage 3 or 4 lung cancer and went on to further investigations. These patients followed the same diagnostic pathway as the prehabilitation patients. Anyone admitted to hospital or deemed for best supportive care at their new patient respiratory appointment was excluded from the historical control cohort. Case files were not individually matched. Approximately 60% of patients with stage 3 and 4 lung cancer in these time periods were deemed appropriate as historical controls, which matched the number of patients eligible from prehabilitation.

The backbone of the analysis was derived from routine data from the lung cancer audit for NHS Lothian, and the historical cohort was identified from this data set. Electronic patient records, chemotherapy, radiotherapy records and Public Health Scotland data allowed for an analysis of patient-specific outcomes. Date of diagnosis was standardised as per local audit data, with a consistent definition between historical controls and prehabilitation patients. Date of diagnosis related to the date of the first test leading to a diagnosis of lung cancer. Admission and survival data were collected retrospectively when the prehabilitation project was complete for both the prehabilitation patients and historical controls.

### Analysis Plan

The primary outcomes were length of hospital stay during 6 weeks and survival at 6 months post-date of diagnosis. Exploratory analyses more thoroughly assessed the impact of prehabilitation on oncology services.

Admissions analysis: The admissions analysis assessed the number of accident and emergency (A&E) attendances, inpatient admissions and associated length of stay for the prehabilitation and historical cohorts. If a patient had an A&E attendance or inpatient admission between 0 and 42 days after the date of diagnosis (6 weeks), it was included as an event. This was a pre-specified analysis. Planned admissions, including day-case admissions, were not included in this analysis. We also assessed A&E attendances and hospital admissions in the 30 days and 14 days before death. As the cohorts were of different sizes, these results were expressed as an outcome per 100 patients.

Statistical tests: To compare age and gender between the historical control and prehabilitation patient groups, appropriate statistical tests were chosen based on data distribution and counts. Age was compared using the Wilcoxon Rank-Sum test because the age data was not normally distributed. Categorical variables, such as gender, were compared using Pearson’s Chi-squared test or Fisher’s exact test when expected cell counts were low.

Survival analysis: A pre-specified survival analysis was conducted using the Kaplan–Meier (KM) method to estimate and compare survival probabilities between the prehabilitation and historical cohorts. Follow-up time (in years) was calculated from the diagnosis date to the date of death or the date of censoring. To assess survival differences at specific early time points, analyses were performed by censoring patient data at 3 months (0.25 years), 6 months (0.5 years) and 12 months (1.0 years) post-diagnosis date. This was deemed the most appropriate method of assessing mortality given the early- and short-term nature of the intervention. For each analysis, a separate KM model was fitted using the corresponding censored time-to-event data. Survival curves were generated for each cohort, and differences between the curves were statistically evaluated using the log-rank test. General Register Office death data was used with a cut-off date of the 31st March 2025.

Blood analysis: An exploratory analysis of background inflammation using routine blood tests was performed. Scottish Immunotherapy Prognostic Score (SIPS) was calculated using neutrophil count and albumin [21]. Blood samples were included for a month (31 days) before and after diagnosis, but before initiation of any anti-cancer treatment.

The Scottish Inflammatory Prognostic Score (SIPS) categorises patients based on albumin and neutrophil count. A score of 0 (low risk) is assigned if albumin is ≥35 g/L and the neutrophil count is ≤7.5 × 10^9^/L. A score of 1 (moderate risk) is given when either albumin is <35 g/L or the neutrophil count is >7.5 × 10^9^/L (but not both criteria are met). A score of 2 (high risk) indicates that both albumin is <35 g/L and the neutrophil count is >7.5 × 10^9^/L [22].

## 3. Results

### 3.1. Demographics

A total of 103 patients attended the intervention across both time periods. Six patients were excluded, five due to a diagnosis of stage 1 or 2 lung cancer, and one due to an alternative malignant diagnosis. Ninety-seven prehabilitation patients were included in this analysis. One historical control was removed as their diagnosis was based on a CXR alone. The patients’ demographics are reported in Table 1. There were no statistically significant differences in either the age distributions (Wilcoxon Rank-Sum test, *p* = 0.739) or gender distributions (Pearson’s Chi-squared test, *p* = 0.280) between the historical and prehabilitation cohorts

### 3.2. Prehabilitation

The median time to the respiratory clinic was 15 days for the historical controls and 16 days for the prehabilitation cohort from the date of diagnosis. The median time to prehab was 25 days from the date of diagnosis and 10 days from the new patient respiratory clinic. Overall, 72% of the patients attended all three interventions. Individual attendance rates were 95% (palliative care), 85% (dietetics) and 77% (physiotherapy).

### 3.3. Hospital Admissions and Length of Stay

**Figure 2:**
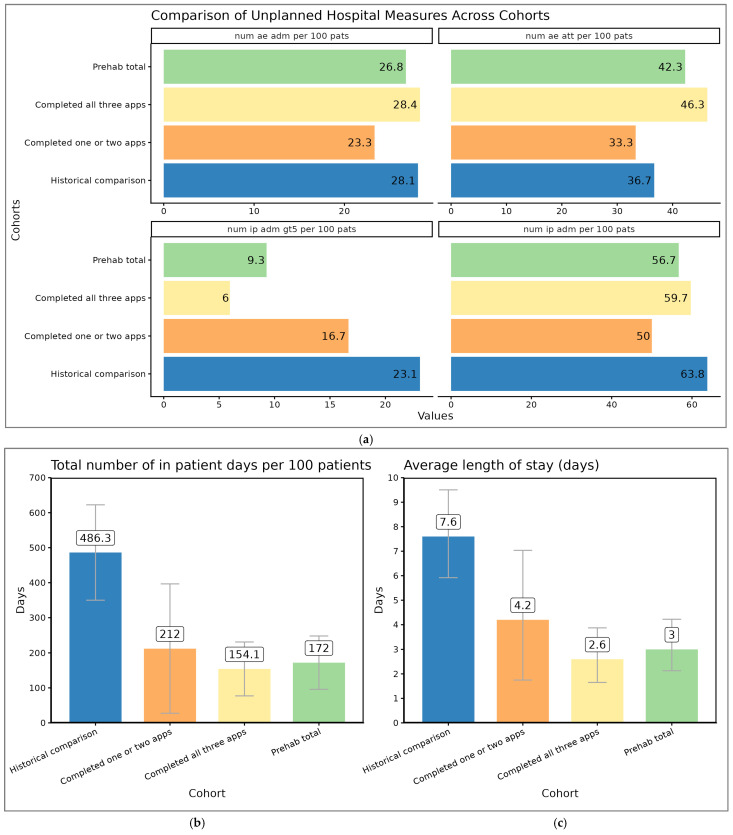
(**a**) A&E attendances, A&E admissions, admissions overall and number of admissions that lasted more than 5 days. A&E attendance, A&E admissions and admissions were broadly similar. The number of admissions lasting longer than 5 days was considerably lower in the prehabilitation group than in the control group (9.3 admissions vs. 23.1 admissions). (**b**) Time spent in hospital per 100 patients, which is lower in patients receiving prehabilitation. (**c**) The average length of stay per admission, which is shorter in patients receiving prehabilitation.

The time spent in hospital was lower in the prehabilitation group when compared to the historical controls. This was a trend in all-comers; however, LOS was statistically significantly shorter in those who were admitted (*p* = 0.0014, Wilcoxon Rank-Sum test). Length of stay (LOS) was significantly less in the prehabilitation cohort who completed all three prehabilitation appointments, when compared to historical controls (2.6 days vs. 7.6 days, a reduction of 66%). The patients who completed all three prehabilitation appointments demonstrated the lowest rates of prolonged admissions per 100 patients (>5 days: *n* = 6.0), shortest average LOS per admission (2.6 days) and lowest total LOS per 100 patients (154.1 days), showing greater reductions compared to both the historical cohort (*n* = 23.1, 7.6 days, 486.3 days, respectively) and those who completed only one or two appointments (*n* = 16.7, 4.2 days, 212.0 days, respectively), as shown in Figure 2.

Initially, a Wilcoxon Rank-Sum test was performed to compare the overall distribution of LOS between the prehabilitation and historical cohorts, encompassing all patients in each group. This did not reveal a statistically significant difference in LOS (*p* = 0.053), but there was a trend towards shorter admissions. The 95% confidence interval ranged from −4.60 × 10^−5^ to 5.03 × 10^−5^.

To specifically investigate differences in LOS duration among patients who were admitted to hospital, a subsequent analysis was conducted by excluding patients with no recorded unplanned inpatient stay. When comparing only those patients who were admitted, the Wilcoxon Rank-Sum test showed a statistically significant difference in LOS between the prehabilitation and historical cohorts (*p* = 0.0014). The 95% confidence interval indicated that the historical cohort’s median LOS was significantly higher than the prehabilitation cohort’s median LOS by an estimated 0.95 to 5.73 days. These findings suggest that while there was no overall significant difference in the tendency for any length of stay (including no stay) between the cohorts, a significant difference emerged when focusing on admitted patients. The historical cohort experienced longer hospital stays for those individuals who were admitted, compared to the patients admitted in the prehabilitation cohort.

#### 3.3.1. Impact of Length of Stay by Treatment

**Figure 3:**
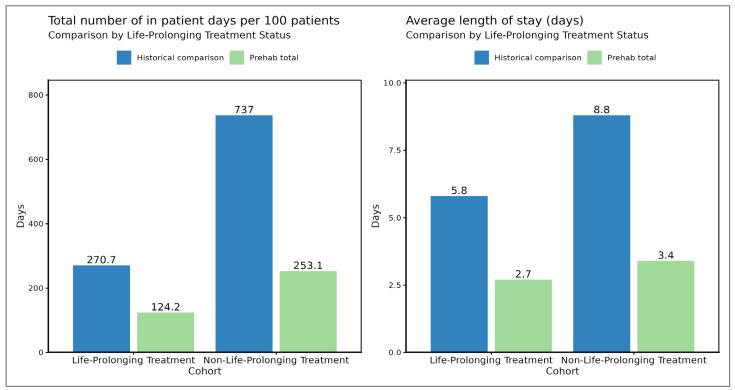
Impact of prehabilitation for patients sub-divided into those who received life-prolonging treatment (SACT, radical RT and high-dose palliative RT) and those who did not. Prehabilitation appears to reduce time spent in hospital, irrespective of whether patients went on to receive life-prolonging treatment.

The average length of stay per admission was lower for the patients in the prehabilitation cohort compared to those in the historical cohort, regardless of whether life-prolonging treatment was given (2.7 vs. 5.8 days, respectively) or not (3.4 vs. 8.8 days, respectively). Non-life-prolonging treatment was associated with a longer average LOS per admission compared to life-prolonging treatment (Figure 3).

Prehabilitation was associated with a decrease in total inpatient days per 100 patients for both treatment groups. The reduction was particularly pronounced for the patients receiving non-life-prolonging treatment (a decrease of 483.9 days per 100 patients, from 737 to 253.1 days), while a considerable reduction was also observed for those receiving life-prolonging treatment (a decrease of 146.5 days per 100 patients, from 270.7 to 124.2 days).

#### 3.3.2. Time to Inpatient Admission from Diagnosis

The mean time to first unplanned inpatient (IP) admission following diagnosis was 87.4 days (prehabilitation) and 97.0 days (historical). However, due to the potential for outlier observations to distort the mean, median times were compared. The median time to first IP admission was substantially longer in the prehabilitation cohort (43 days) than in the historical cohort (34 days), suggesting that the prehabilitation patients generally experienced a longer period without an unplanned hospital stay after diagnosis.

#### 3.3.3. Admissions Within 14 and 30 Days of Death

**Figure 4:**
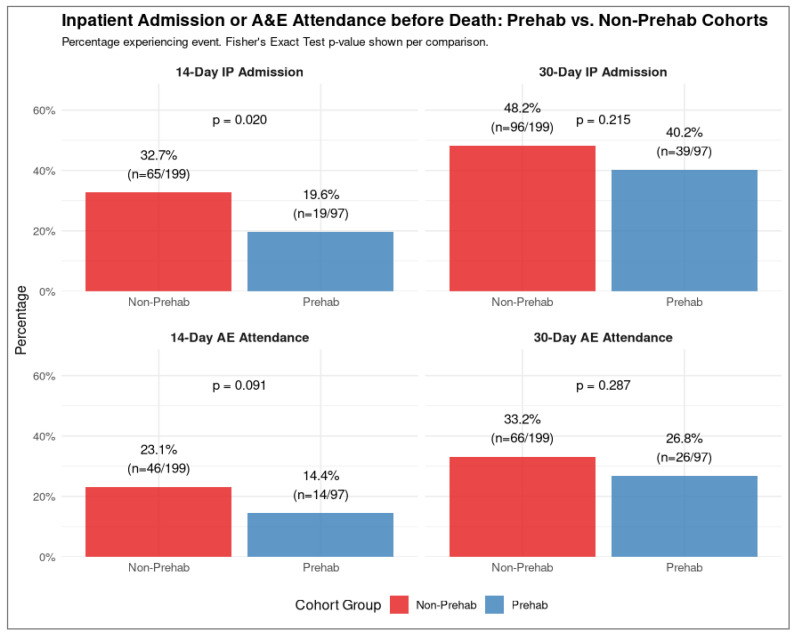
IP admissions and A&E attendances of patients receiving prehabilitation vs. historical controls. There were fewer admissions and A&E attendances 30 days and 14 days before death. There was a statistically significant reduction in admissions 14 days before death, when using Fisher’s exact test.

Examination of unplanned hospital resource utilisation prior to death indicated potentially reduced usage by the prehabilitation cohort compared to the non-prehabilitation cohort. IP admissions within 30 days of death were lower in the prehabilitation group, although this difference was not statistically significant (40.2% vs. 48.2%, *p* = 0.215). However, this difference reached statistical significance within the 14 days preceding death (Figure 4). The prehabilitation group had significantly lower odds of IP admission compared to the non-prehabilitation group during this shorter timeframe (19.6% vs. 32.7%; OR 0.50, 95% CI [0.26, 0.92], *p* = 0.020). A&E attendances also showed lower rates in the prehabilitation cohort, but these differences did not achieve statistical significance at either 30 days (26.8% vs. 33.2%, *p* = 0.287) or 14 days (14.4% vs. 23.1%, *p* = 0.091) before death.

### 3.4. Treatment Rates

The proportion of patients with stage 3 cancer receiving treatment was similar. There was an increase in the patients with stage 4 disease receiving SACT from 25.6% to 42.3% (see Table 2). The number of stage 3 patients receiving radical radiotherapy decreased (15 vs. 1 patient, 7.5% vs. 1% of the cohorts), but numbers in both cohorts were small.

### 3.5. Inflammatory Status

**Figure 5:**
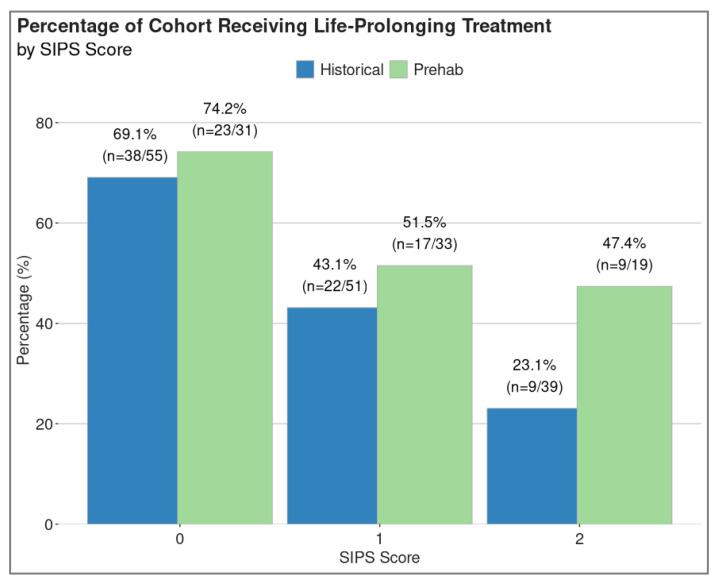
Comparison of treatment rates by inflammatory status using the Scottish Immunotherapy Prognostic Score (SIPS). Treatment rates appear to be higher in those with low, medium and high levels of inflammation.

It was possible to calculate the SIPS score for 73% (*n* = 145) of the patients in the historical cohort and 86% (*n* = 83) of the patients in the prehabilitation cohort. Life-prolonging treatment rates increased in all patient groups in the prehabilitation cohort, with the largest change in those with the highest scores (and, therefore, the highest levels of inflammation), as shown in Figure 5.

### 3.6. Survival Analysis

**Figure 6:**
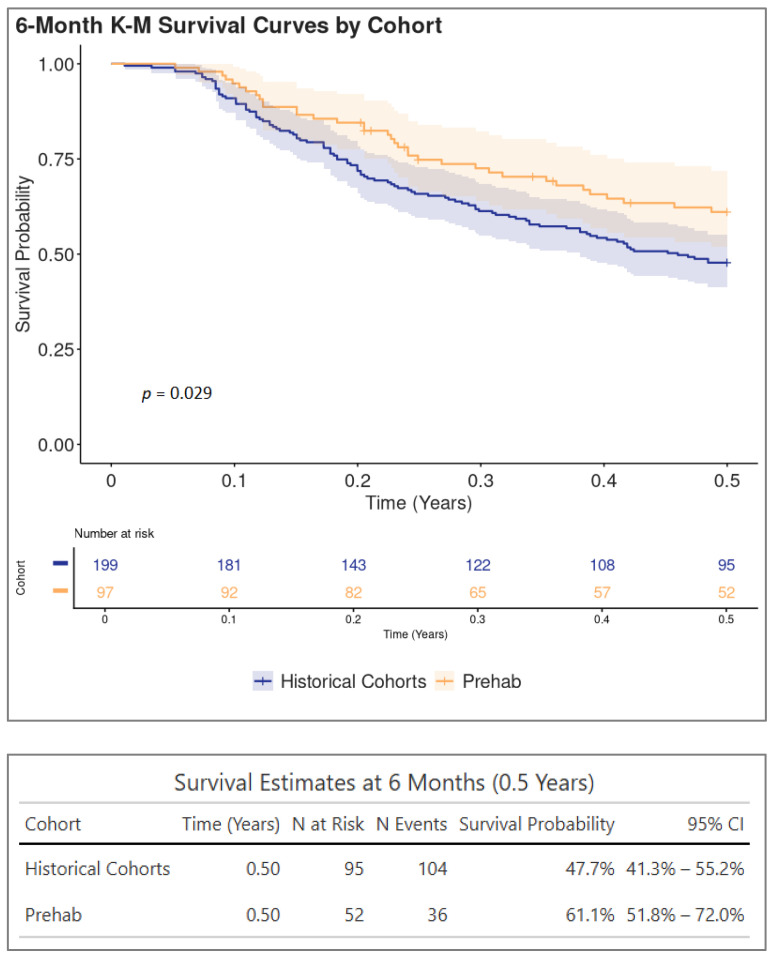
Kaplan–Meier analysis of survival at 6 months. There was a statistically significant survival benefit at 6 months (*p* = 0.029, 61.1% in the prehabilitation group vs. 47.7% in the historical controls).

Prehabilitation appeared to improve short-term survival in this cohort. The Kaplan–Meier analysis comparing survival over the first 6 months (0.5 years) between the historical and prehabilitation cohorts is presented in Figure 6. The overall survival distributions differed between the groups (*p* = 0.029), with the prehabilitation cohort demonstrating higher survival probability throughout the period. At 6 months, the estimated survival probability was 61.1% (95% CI: 51.8–72.0%) for the prehabilitation cohort, compared to 47.7% (95% CI: 41.3–55.2%) for the historical cohort. While the 95% confidence intervals for the survival estimates overlap at this specific 6-month time point, the significant log-rank test indicates a difference in the overall survival experience accumulated across the entire 6-month follow-up period. There were differences in survival at 3 months (65.8% vs. 74.8% *p* = 0.091) and 12 months (31.7% vs. 40.3%, *p* = 0.061) between the historical controls and the prehabilitation patients, but these were not statistically significant.

## 4. Discussion

Early prehabilitation shows a likely trend in reducing the time spent in hospital for all patients who were investigated for locally advanced and metastatic lung cancer. This appears to occur regardless of whether those patients go on to receive active treatment. Prehabilitation appears to increase SACT treatment rates in those with stage 4 disease and reduce the proportion of patients receiving best supportive care.

There was a reduction in the time spent in hospital of 61% (mean 7.6 days to mean 3 days, *p* = 0.053) in all-comers, and LOS was statistically significantly reduced when the admitted prehabilitation patients were compared to the admitted historical cohorts (*p* = 0.0014, 95% CI 0.95 to 5.73 days). Prehabilitation reduced the time spent in hospital by 314 days per 100 patients, or by 3.1 days per patient undergoing prehabilitation (see Figure 3).

The patients who received all three interventions had a greater reduction in the time spent in hospital when compared to those who received one or two interventions (reduction of 66% vs. 45%).

In addition to reducing the time spent in hospital, early prehabilitation appeared to affect short-term survival. There was a trend towards a higher survival rate at 3 months, 6 months and 12 months from the date of diagnosis. There was a statistically significant improvement in survival at 6 months (*p* = 0.029, 61.1%, 95% CI: 51.8–72.0% for the prehabilitation cohort compared to 47.7%, 95% CI: 41.3–55.2% for the historical cohort). Our initial cohort showed an increased median survival. This may suggest that a longer follow-up shows a more sustained survival benefit.

This is a real-world data comparison rather than a randomised trial. Whilst there were no obvious differences in gender, age or disease staging, it is important to recognise possible confounding factors, such as selection bias and the influence of the COVID-19 pandemic. The outcomes of “fit” patients with newly diagnosed lung cancer are very varied. Some patients may rapidly deteriorate and require urgent symptom control. Others may proceed to a much better outcome. Because the intervention is so early in the pathway, we accounted for this variation by identifying a large cohort of historical controls who followed the same pathway as the prehabilitation patients. The time from diagnosis to the respiratory clinic was very similar in both populations (15 vs. 16 days).

Despite possible limitations, however, our findings are supported by existing data. The most well-known study, conducted by Temel et al., showed that early palliative care improved survival in patients with lung cancer who were due to start SACT [23]. A recent meta-analysis identified six further randomised studies (including 1200 patients), which assessed the efficacy of early palliative care in patients with NSCLC [24]. In total, three studies reported OS, with a pooled analysis suggesting improved survival for the group receiving early palliative care (HR 1.60 (95% confidence interval (CI) of 1.22–1.98). Five studies reported improved quality of life using FACT scores (pooled standardised mean difference = 1.18, 95% CI of 1.04–1.31). Our project builds on these results and suggests that multiple interventions further improve outcomes when compared to patients receiving one or two out of the three available interventions.

The role of supportive care in patients with poor-prognosis cancers is established. In a wider English project, enhanced supportive care was shown to reduce admissions, improve symptom control, improve quality of life, reduce 30-day mortality from chemotherapy, reduce healthcare costs and improve overall survival [25,26]. A qualitative assessment in this population identified benefits to patients in terms of their quality of life [27]. Even in the context of palliative care physicians, supportive care is now a formal part of palliative care training in the UK [28].

A recent review suggested that the intervention point for early palliative care can be defined in terms of time from diagnosis, prognosis of diagnosis, location-based (e.g., inpatient or outpatient) and severity of symptoms [29]. The EPIC project intervention seems appropriate in terms of high symptom burden, poor prognosis and early intervention time point. Palliative care is evolving with the development of rehabilitative palliative care, aiming to enable patients to live to their fullest until they die [30].

This raises the question of how these interventions directly benefit the patient. Early aggressive symptom control may prevent de-conditioning of the patient. Effective symptom control, e.g., for pain or nausea, may limit its impact on nutrition or physical activity. The palliative care intervention allowed time for introducing better health literacy and safety netting for symptoms. This meant that patients potentially had a greater understanding of what to do in terms of managing their symptoms, e.g., more effective use of short-acting strong opiates for breakthrough pain in a patient already on long-acting opiates. Our hypothesis is that better symptom control allowed the patients to subsequently benefit more from expert advice on nutrition and physical activity. There is an overwhelming amount of information on diet and cancer available online, much of which is not evidence-based. The key information can be streamlined and individualised for patients, particularly if they have specific dietary needs. The same is true for physiotherapy. Many patients are very breathless. A strategy to manage physical activity in a less fit patient can be developed by an expert. In summary, it is likely that the observed benefits are based on expert, individualised advice. This minimises the loss of fitness across the diagnostic pathway, leading to an increased chance of treatment. Further qualitative research is required to better understand these complex interactions.

The benefits of independent elements of prehabilitation are less clear. Our data suggests that an increased benefit was seen in the patients receiving all three interventions. This means they needed to be well enough to attend all three appointments. A recently published UK study showed that an aggressive exercise intervention led to an improvement in physical fitness for patients undergoing curative radiotherapy [31]. The patients were divided into those receiving supervised and unsupervised exercise. The number of patients attending three exercise sessions a week increased from 16% to 52%, with a statistically significant improvement in the 6 min walk test. Dietetic benefits are harder to prove. A previous study at the Royal Marsden failed to show benefits, but adherence to supplements was low [32].

Tri-modality prehabilitation is a series of complex interactions. The effects of the interventions on SACT rates are less clear. Active treatment rates in patients with stage 4 lung cancer improved from 27.6% to 44.3%, which is an encouraging result. Overall, the number of patients receiving best supportive care reduced from 45.2% to 27.8%. The number of patients with stage 3 lung cancer receiving SACT was stable; however, the radical radiotherapy rate declined. However, the number of eligible patients for this treatment was very small in both groups. The prehabilitation cohort contained fewer patients with stage 3a disease than the historical control cohort (3.1% vs. 13.1%) but similar proportions of patients with stage 3b and 3c disease. It is possible that fewer stage 3 patients in the prehabilitation cohort were suitable for radical treatment, but further data and larger-scale studies are required to understand this relationship. As a confounder, previously published English data suggested there is huge variation in radical treatment rates in stage 3 NSCLC between hospitals, ranging from 8 to 80%, potentially due to differences in opinion amongst oncologists about what bulk of disease is radically treatable [33].

This multidisciplinary prehabilitation project began as a pilot project. It used real-world clinical data to assess outcomes, meaning the results are reflective of routine clinical practice. An initial challenge was how to measure outcomes in a way that could be extracted from both the historical and prehabilitation cohorts. As a result, we were unable to collect detailed information about the patients’ quality of life. The project also experienced a pause during a gap in funding, when the prehabilitation clinic stopped for approximately 6 months. Interestingly, we found that time spent in hospital increased during that gap in prehabilitation service and then decreased again when prehabilitation restarted (Supplementary Materials, Figure S1) We suggest that this change strengthens the argument that there is a beneficial association between the intervention and outcome.

One of the questions generated by this work is whether we need to provide the same level of prehabilitation to every patient. This project saw reductions in the time spent in hospital in both the patients receiving life-prolonging treatment and those who did not. However, approximately a quarter of the patients did not receive all three interventions. Further work is required to understand the reasons for this and the potential impact. The sensitivity analysis suggests that the combination of all three interventions is important. In the first iteration of this project, we delivered all three interventions at the same appointment. This was overwhelming for the patients, so subsequent appointments were spread across 2 weeks: in week 1, the patients saw the palliative/supportive care physician, and in week 2, the patients saw the physiotherapist and dietitian. Anecdotally, some patients became too unwell to attend the second week of the intervention, so they may still have benefited from the early palliative care intervention in week 1.

Routine blood tests reflective of the systemic inflammatory response may offer a method of prognosticating patients and defining red, orange and green patients [34]. This project demonstrated a trend toward increasing treatment rates across all three groups of low, medium and high levels of systemic inflammation, using the SIPS [22]. The greatest improvement was seen in the patients with the highest levels of systemic inflammation (i.e., SIPS2), suggesting the benefit of prehabilitation is widespread, rather than in a specific prognostic group. However, the fact that blood tests were not available for the whole cohort was a limitation and may have influenced the results.

Our work demonstrates that multidisciplinary prehabilitation has the potential to reduce hospital bed/days. Cost savings were not explored in detail in this work. The direct staffing cost for the project was approximately GBP 50,928 per annum, with the capacity to provide interventions for 120 patients, defined as 3 patients per week over a 40-week year (see Supplementary Materials, Table S1). The direct cost was GBP 424 per patient. An internal analysis suggested that each 24 h bed/day that was prevented had a cost saving of approximately GBP 400; however, the only referenceable cost that could be identified for an acute medical bed was GBP 800 per 24 h bed/day [35]. If each patient receiving tri-modaility prehabilitation saves 3 days in hospital, this equates to a saving of GBP 776 to GBP 1976 per patient. If 60% of stage 3 and 4 patients are fit for prehabilitation, this would give an eligible population of nearly 2000 patients per annum (see Supplementary Materials, Table S2), equating to a potential saving of 6000 bed/days and GBP 1,552,000 to GBP 3,952,000 per annum Scotland-wide. This cost-saving is potentially significant, but it avoids costs; it does not release funding. It reduces pressure on unscheduled care, but it does not avoid specific costs. More detailed health economic analyses would be beneficial. This is an inexpensive intervention that runs in parallel with the standard patient pathway, which has been identified as a challenge in other prehabilitation projects [36]. The intervention could also be available in a small District General Hospital as well as a specialist Cancer Centre.

Further work is needed to understand the effect of prehabilitation on treatment intensity and duration. This work will inform the planning of future clinical trials. It will help with more formal statistical work, such as sample size calculations. Our aim going forward is to test this hypothesis in a bigger, more formal study, including patients with other less survivable cancers, such as pancreatic cancer. We also aim to collaborate with local third-sector/charity organisations to establish an ongoing prehabilitation/rehabilitation continuum by integrating other elements of care, such as ongoing exercise interventions.

## 5. Conclusions

Early multi-modal prehabilitation seems to reduce hospitalisations and may improve survival and treatment uptake in lung cancer patients. These findings should be confirmed in a formal randomised clinical trial. Further qualitative work is also required to understand the complex interactions and benefits of these interventions.

## Figures and Tables

**Table 1:** Patient and treatment characteristics by cohort. SIPS: Scottish Inflammatory Prognostic Score [22].

Category	Sub-Category	Historical Controls Number (%)	Prehabilitation Number (%)
Number of pts		199	97
Age (median, range)		70 (34–91)	68 (38–89)
Gender	Male	101 (50.8%)	42 (43.3%)
	Female	98 (49.2%)	55 (56.7%)
Diagnosis	Radiological (no biopsy)	25 (12.6%)	7 (7.2%)
	Small cell lung cancer	48 (24.1%)	17 (17.5%)
	NSCLC	126 (63.3%)	72 (74.2%)
	Mesothelioma	0 (0%)	1 (1%)
	No. of patients with stage 3 disease	72 (36%)	30 (31%)
	No. of patients with stage 4 disease	127 (64%)	67 (69%)
Inflammatory markers, SIPS	% Bloods available for SIPS	145 (73%)	83 (86%)
	% SIPS 0	55 (37.9%)	31 (37.3%)
	% SIPS 1	51 (35.2%)	33 (39.8%)
	% SIPS 2	39 (26.9%)	19 (22.9%)

**Table 2:** Identified treatment rates. Exploratory analysis of treatment rates in the historical controls vs. the prehab cohort. There is a trend towards increased SACT treatment rates in patients with stage 4 disease. SACT: Systemic anti-cancer drug treatment, RT: radiotherapy, BSC: best supportive care. Life-prolonging treatment is defined as SACT, radical radiotherapy or high-dose palliative radiotherapy (i.e., all treatment groups except low-dose palliative radiotherapy and BSC).

		Historical Controls (199 Patients)	Prehabilitation Cohort (97 Patients)
Treatment	% stage 3 receiving SACT	31 (15.6%)	16 (16.5%)
	% stage 4 receiving SACT	51 (25.6%)	41 (42.3%)
	% stage 3 receiving radical RT (55–60 Gy in 20–30 doses)	15 (7.5%)	1 (1%)
	% stage 3 and 4 receiving high-dose palliative radiotherapy (36–39 Gy in 12–13 doses)	7 (3.5%)	2 (2.1%)
	% stage 3 and 4 receiving palliative radiotherapy (8–20 Gy in 1–5 doses)	2 (1.0%)	9 (9.3%)
	% stage 3 and 4 BSC	90 (45.2%)	27 (27.8%)
	% stage 3 patients receiving life-prolonging treatment	52 (26.1%)	18 (18.6%)
	% stage 4 patients receiving life-prolonging treatment	55 (27.6%)	43 (44.3%)

## Data Availability

The data presented in this study are available in this article (and Supplementary Material).

## Supplementary Materials

The following supporting information can be downloaded at: https://www.mdpi.com/article/10.3390/cancers17203329/s1, Figure S1: Figure showing admissions by prehab cohort when the project started and stopped. Period (a) Historical controls 1, (b) Prehabilitation 1, (c) Historical controls 2, (d) Prehabilitation 2; Table S1: Table of direct staffing costs for prehabilitation project. Updated for 2025/26. Costs include additional staffing costs. https://www.gov.scot/publications/nhs-staff-pay/; Table S2: Table of number of patients eligible for prehabilitation Scotland wide.
