# Supplementary material for: Multidisciplinary Prehabilitation Reduces Hospitalization Time and Suggests Improved Survival in Patients with Radiologically Diagnosed Lung Cancer
Source: Cancers (Basel). 2025 Oct 15;17(20):3329. doi: 10.3390/cancers17203329 (PMC12562606; doi:10.3390/cancers17203329)
Supplement: Supplementary file 1 [file cancers-17-03329-s001.zip › cancers-3751564-supplementary.pdf]

Supplementary materials

Figure S1. Figure showing admissions by prehab cohort when the project started and stopped. Period a) Historical controls 1, b) Prehabilitation 1, c) Historical controls 2, d) Prehabilitation 2.

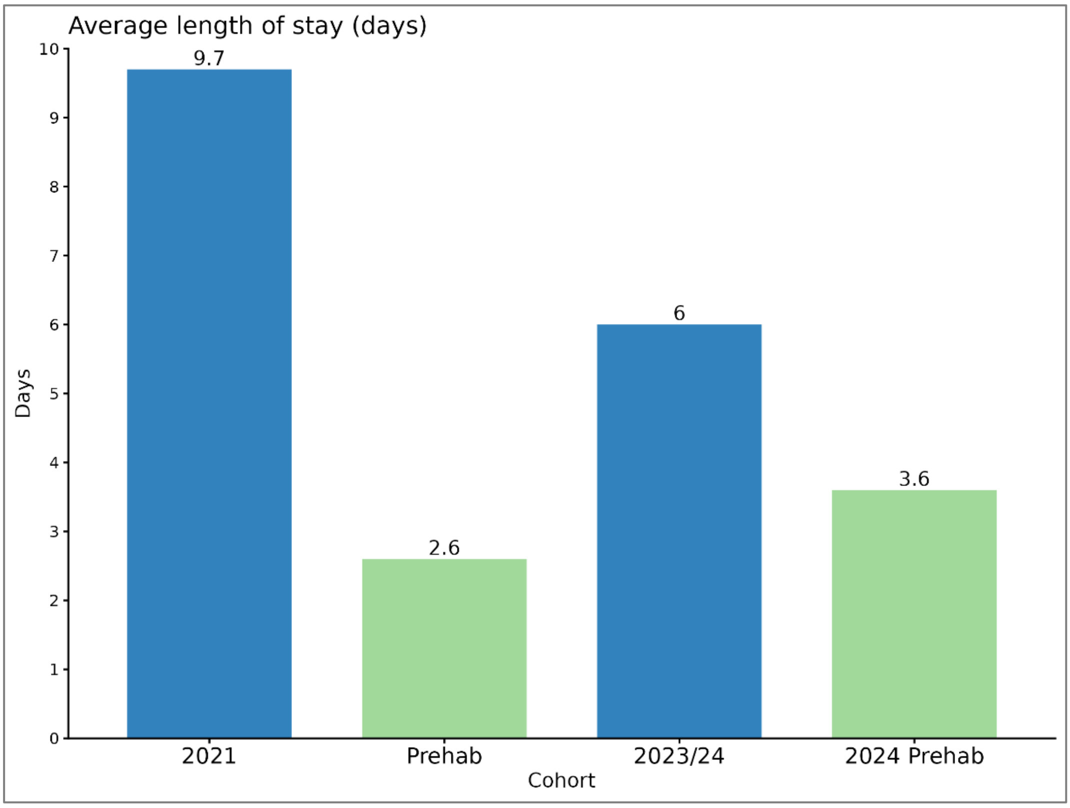

Table S1. Table of direct staffing costs for prehabilitation project. Updated for 2025/26. Costs include additional staffing costs. https://www.gov.scot/publications/nhs-staff-pay/

		Whole time equivalent	Cost per annum (£)	Cost for project (£)
Oncology dietitian	Band 6	0.20	65,879	13,175.80
Pulmonary rehab physiotherapist	Band 6	0.20	65,879	13,175.80
Consultant palliative care consultant	n/a	0.1	170,258	17,026
Admin support	Band 2	0.20	37,752	7550.4
Total				50,928

Table S2. Table of number of patients eligible for prehabilitation Scotland wide

Cancer Network/ Healthboard	No of stage 3 lung pts/year[1]	No of stage 4 lung pts/year[1]	No of pts appropriate for prehab (60%)[2]	No of hospital bed days freed up through prehab/year
**NCA**	**229**	**564**	**476**	**1475**
NHS Grampian	67	199	160	**495**
NHS Highland	52	138	114	**353**
NHS Orkney	1	7	5	**15**
NHS Shetland	1	6	4	**13**
NHS Tayside	99	202	181	**560**
NHS Western Isles	9	12	13	**39**
**SCAN**	**293**	**551**	**506**	**1570**
NHS Borders	17	47	38	**119**
NHS Dumfries & Galloway	26	57	50	**154**
NHS Fife	72	150	133	**413**
NHS Lothian	178	297	285	**884**
**WOSCAN**	**500**	**1074**	**944**	**2928**
NHS Ayrshire & Arran	75	163	143	**443**
NHS Forth Valley	56	125	109	**337**
NHS Greater Glasgow & Clyde	248	487	441	**1367**
NHS Lanarkshire	121	299	252	**781**
**Total Scotland**	**1022**	**2189**	**1927**	**5972**

1. https://publichealthscotland.scot/publications/cancer-staging-data/2022-cancer-staging-data-28-november-2023/

2. https://spcare.bmj.com/content/early/2024/04/17/spcare-2024-004869.share
